# Design and Evaluation of Pegylated Large 3D Pore Ferrisilicate as a Potential Insulin Protein Therapy to Treat Diabetic Mellitus

**DOI:** 10.3390/pharmaceutics15020593

**Published:** 2023-02-09

**Authors:** B. Rabindran Jermy, Mohammed Salahuddin, Gazali Tanimu, Hatim Dafalla, Sarah Almofty, Vijaya Ravinayagam

**Affiliations:** 1 Department of NanoMedicine Research, Institute for Research and Medical Consultations (IRMC), Imam Abdulrahman Bin Faisal University, P.O. Box 1982, Dammam 31441, Saudi Arabia; 2 Department of Clinical Pharmacy, Institute for Research and Medical Consultations (IRMC), Imam Abdulrahman Bin Faisal University, P.O. Box 1982, Dammam 31441, Saudi Arabia; 3 Center for Refining and Advanced Chemicals, Research Institute, King Fahd University of Petroleum and Minerals, P.O. Box 5040, Dhahran 31261, Saudi Arabia; 4 Core Research Facilities (CRF), King Fahd University of Petroleum and Minerals, P.O. Box 613, Dhahran 31261, Saudi Arabia; 5 Department of Stem Cell Research, Institute for Research and Medical Consultations (IRMC), Imam Abdulrahman Bin Faisal University, P.O. Box 1982, Dammam 31441, Saudi Arabia; 6 Deanship of Scientific Research & Department of NanoMedicine Research, Institute for Research and Medical Consultations (IRMC), Imam Abdulrahman Bin Faisal University, P.O. Box 1982, Dammam 31441, Saudi Arabia

**Keywords:** ferrisilicate, PEG, insulin, encapsulation, diabetic mellitus

## Abstract

An iron-based SBA-16 mesoporous silica (ferrisilicate) with a large surface area and three-dimensional (3D) pores is explored as a potential insulin delivery vehicle with improved encapsulation and loading efficiency. Fe was incorporated into a framework of ferrisilicate using the isomorphous substitution technique for direct synthesis. Fe^3+^ species were identified using diffuse reflectance spectroscopy. The large surface area (804 m^2^/g), cubic pores (3.2 nm) and insulin loading were characterized using XRD, BET surface area, FTIR and TEM analyses. For pH sensitivity, the ferrisilicate was wrapped with polyethylene glycol (MW = 400 Daltons) (PEG). For comparison, Fe (10 wt%) was impregnated on a Korea Advanced Institute of Science and Technology Number 6 (KIT-6) sieve and Mesocellular Silica Foam (MSU-F). Insulin loading was optimized, and its release mechanism was studied using the dialysis membrane technique (MWCO = 14,000 Da) at physiological pH = 7.4, 6.8 and 1.2. The kinetics of the drug’s release was studied using different structured/insulin nanoformulations, including Santa Barbara Amorphous materials (SBA-15, SBA-16), MSU-F, ultra-large-pore FDU-12 (ULPFDU-12) and ferrisilicates. A different insulin adsorption times (0.08–1 h), insulin/ferrisilicate ratios (0.125–1.0) and drug release rates at different pH were examined using the Korsmeyer–Peppas model. The rate of drug release and the diffusion mechanisms were obtained based on the release constant (k) and release exponent (n). The cytotoxicity of the nanoformulation was evaluated by 3-(4,5-dimethylthiazol-2-yl)-2,5-diphenyltetrazolium bromide (MTT) assay using human foreskin fibroblast (HFF-1) cells. A low cytotoxicity was observed for this nanoformulation starting at the highest concentrations used, namely, 400 and 800 μg. The hypoglycemic activity of insulin/ferrisilicate/PEG on acute administration in Wistar rats was studied using doses of 2, 5 and 10 mg/kg body weight. The developed facile ferrisilicate/PEG nanoformulation showed a high insulin encapsulation and loading capacity with pH-sensitive insulin release for potential delivery through the oral route.

## 1. Introduction

Diabetic mellitus (DM) is a metabolic disorder categorized by hyperglycemia due to inopportune insulin secretion. DM is differentiated as type I (insulin-reliant) and type II diabetes (insulin resistance) [1,2]. Diabetic mellitus, a disease termed as a life style disease, is quickly turning into a global epidemic. The prime reason for this is attributed due to change in life style, unhealthy diets and lack of awareness. In 2019, diabetic severity resulted in 1.5 million mortalities, and notably, 48% of these deaths occurred before 70 years of age. Among the two types of diabetes, type II diabetes is dominant, accounting for 95% (WHO). Recent data show that worldwide, about 537 million people have diabetes, and this number is expected to reach 783 million by 2045 [3]. Such a high percentage and increasing rate of DM is primarily attributed to obesity and changes in life style. The healthcare expenditure spent on diabetic treatment was estimated to be USD 966 billion in 2021.

Insulin was discovered by Frederick Banting in 1921, while Charles Best developed the clinical use of insulin in 1922 [4]. Insulin is administered to control the blood glucose level. Insulin helps to uptake glucose by binding with the insulin receptor and intitiating several protein activations cascades (e.g., Glut-4 transporter to plasma member, influx of glucose, synthesis of glycogen, glycolysis and triglyceride production). Type 1 DM (due to defective pancreatic β cells) depends on the lifelong supply of insulin. In the case of type 2 DM patients, the peripheral cells resist the administration of insulin, while some patients at the lateral life stage also require insulin. In order to treat type 1 DM, a common mode of insulin administration is through the subcutaneous route with about four injections per day. The treatment affects patient compliance and induces several side effects (lipoatrophy). Still, the subcutaneous administration route is preferred due to insulin’s low bioavailability and challenges in developing an effective drug delivery system due to low membrane permeability and molecular size constraints. 

In order to limit the number of injection cycles, a controlled insulin release strategy was followed using zinc and protamine. The used formulation showed poor reproducible kinetic parameters and the effect between meal periods was minimal and led to hypoglycemic events [5]. Accordingly, similar to insulin, insulin detemir and insulin glargine were shown to subvert the hyproglycemic action, but the required dose level was almost double compared to the normal dose of insulin for one day [6]. It has been reported that diabetic mediators similar to insulin could also alter the mitogenic pathway and can be a potential carcinogen in the long run [7]. The construction of a stimuli-responsive smart drug release system is the most recent attractive research direction involving interdisciplinary research between material science and medical science. An appropriately constructed nanovehicle with controlled insulin delivery using biocompatible nanosilica is proposed to overcome the deficiencies in subcutaneous therapy, improve therapeutic efficiency, enhance the stability of drug release and make ease diet control and exercise regiments. Several glucose-sensitive smart drug delivery system based on phenylboronic acid (PBA) and proteins such as concanavalin and glucose oxidase have been reported [8]. However, such a glucose-sensitive, sensor-based nanovehicle requires a multi-step synthesis procedure, the use of solvents and an advanced chemical set-up.

Recently, a biocompatible drug delivery system based on structured silica/polymeric nanocomposites are shown to be a promising nanovehicle to carry insulin [9]. A microneedle design based on mesoporous silica capped with zinc oxide in the form of an insulin reservoir has been reported to effectively control insulin delivery for prolonged periods of time [10]. Several studies are ongoing to improve the efficacy of protein delivery using mesoporous silica/chitosan and poly(lactic-co-glycolic) acid nanoformulations and improve their permeability [11]. The isomorphous substitution of biocompatible metals such as Fe, Zn, Ti, etc., into the silica framework is gaining importance in biomedical applications [12,13]. The use of Fe cations (Fe^3+^ and Fe^2+^) with particle sizes ranging between 3 and 15 nm is gaining attention in multifunctional therapeutics as contrasting agents for magnetic resonance imaging, in hyperthermia treatments and as drug delivery agents [14]. The presence of iron oxide nanoparticles (FeNPs) favors biocompatibility [15], and as such, they are applied in hyperthermia for their anticancer [16,17] and antibacterial activity [18], as well as in tissue engineering [19]. Previously, we reported a direct hydrothermal synthesis of Iron-incorporated Santa Barbara Amorphous 16 (FeSBA-16) [20]. The presence of large 3D cubic pores of ferrisilicate could be exploited for insulin entrapment/loading capacity and insulin release. The wrapping of the nanocarrier with polyethylene glycol is reported to improve the bioavailability and drug stabilization and facilitate the transport of protein across human gastrointestinal fluid [21]. In this study, we investigated the effect of a pegylated, large 3D porous ferrisilicate/insulin nanoformulation for diabetes management. The textural characteristics are investigated using different physico-chemical characterization techniques. The insulin encapsulation/loading capacity and the pH-based, smart kinetic release behavior in response to stimuli were studied for insulin release. Furthermore, the nanoformulation toxicity in vitro and hypoglycemic effect in vivo were assessed.

## 2. Material and Methods

The silica source tetraethylorthosilicate (reagent grade, 98%, Sigma Aldrich, Darmstadt, Germany) and non-ionic template Pluronic F127 (BioReagent, suitable for cell culture, BASF, Wyandotte, MI, USA), iron(III) nitrate nonahydrate (≥99.95%, BioReagent, suitable for cell culture, Sigma Aldrich, Saint Louis, MO, USA), n-butanol (≥99%, anhydrous, Sigma Aldrich, Saint Louis, MO, USA), human recombinant insulin (rHu, dry powder, Sigma-Aldrich Chemie Holding GmbH, Taufkirchen, Germany) and polyethylene glycol (BioUltra, MW = 400 Daltons, Sigma-Aldrich Chemie Holding GmbH, Taufkirchen, Germany) were obtained from Sigma Aldrich. All chemicals were used as received without any further purification.

### 2.1. Synthesis

#### 2.1.1. Synthesis of Ferrisilicate Using Hydrothermal Technique

Fe-SBA-16, termed as ferrisilicate, was prepared using sol–gel technique. The ferrisilicate containing Fe species can be tuned between SiO_2_/Fe_2_O_3_ ratios of 50 and 250. In the present study, the Fe content can reach a SiO_2_/Fe_2_O_3_ ratio of 50. In brief, 5 g of F127 was dissolved in acidic HCl solution (2 M) and stirred for 1 h. Then, 16 g of n-butanol (co-solvent) was added along with 24 g of tetraethylorthosilicate and the iron source (0.186 g of iron nitrate nonahydrate (Si/Fe ratio 250)) and stirred for 24 h. The mixture was stored in a polypropylene bottle (Nalgene, Thermo Fisher Scientific, NY, USA) and transferred to an oven to be hydrothermally aged at 100 °C for 24 h. The precipitate was filtered, washed several times with excess water and dried at 100 °C for 12 h. The as-synthesized sample was finally calcined at 550 °C for 6 h.

#### 2.1.2. Synthesis of Iron-Impregnated Structured Silica (10 wt% Fe/KIT-6 and 10 wt% Fe/MSU-F) Using Impregnation Technique

Firstly, 0.7235 g of iron nitrate nonahydrate was dissolved in 80 ml of distilled water. Then, 1.0 g of KIT-6, mesosilicalite, or Mesocellular Silica Foam (MSU-F) was added and stirred for 24 h at room temperature (RT). The solution was dried at 120 °C for 3 h and calcined at 500 °C for 2 h.

#### 2.1.3. Insulin/Ferrisilicate

For insulin loading, 80 mg of insulin was added to 8 ml of 0.01 M HCl solution and stirred for 20 min. Then, 160 mg of ferrisilicate was added and stirred at 300 rpm overnight in an ice-cold environment. After that, the mixture was filtered, washed with 5 ml of distilled water and dried at RT (5 h) and stored at 4 °C.

#### 2.1.4. Insulin/Ferrisilicate/PEGylation

For PEGylation, 14 μl of PEG (Molecular weight = 400) was added in 3 ml of deionized water, stirred for 20 min under argon atmosphere and then 150 mg of Insulin/Ferrisilicate was added and stirred under an ice-cold environment for 24 h. Then, the mixture was freeze-dried using the lyophilization technique.

### 2.2. Characterization Techniques

The phase of insulin, insulin/ferrisilicate/PEG, was identified using benchtop XRD (Miniflex 600, Rigaku, Tokyo, Japan). The textural features, including BET surface area, pore size and pore volume, were measured using the nitrogen adsorption technique (ASAP-2020 plus, Micromeritics, Norcross, GA, USA). The ferrite nanoparticles’ chemical coordination was analyzed using DRS-UV-visible spectroscopy analysis (V-750, JASCO, Tokyo, Japan). The insulin functional groups of our nanoformulation were determined using FT-IR spectroscopy (L160000A, Perkin Elmer, Waltham, MA, USA). The morphological variations of insulin/ferrisilicate/PEG were investigated using transmission electron microscopy (TEM, JEM2100F, JEOL, Tokyo, Japan).

### 2.3. Insulin Entrapment Efficiency and Loading Capacity 

Insulin entrapment efficiency (EE %) and loading capacity (LC %) over ferrisilicate were estimated using UV-visible spectroscopy at the specific wavelength of 275 nm.

$$\mathrm{EE}\;\left(\%\right)=\left[\left(\begin{array}{c} \underline{\mathrm{Amount}\;\mathrm{of}\;\mathrm{insulin}\;\mathrm{in}\;\mathrm{ferrisilicate}}\\ \mathrm{Initial}\;\mathrm{amount}\;\mathrm{of}\;\mathrm{insulin} \end{array}\right)\right]\times 100\;\mathrm{LC}\left(\%\right)=\left[\left(\begin{array}{c} \underline{\mathrm{Initial}\;\mathrm{amount}\;\mathrm{of}\;\mathrm{insulin}\;-\mathrm{Insulin}\;\mathrm{in}\;\mathrm{supernatant}}\\ \mathrm{Amount}\;\mathrm{of}\;\mathrm{ferrisilicate}\;+\mathrm{insulin} \end{array}\right)\right]\times 100$$

### 2.4. Insulin Release Study

The release trends of different nanoformulations were studied using the dialysis membrane technique. First, 30 mg of the nanoformulation was placed inside 3 mL of PBS solution inside the dialysis membrane. The release of insulin was monitored under different pH solutions (7.4, 6.8 and 1.2) at 37 °C. At regular time intervals, 10 mL of solution was withdrawn and replaced with an equal volume of fresh solution. The amount of released insulin was identified at a specific wavelength of 275 nm. In order to measure the insulin release, a standard curve for insulin was established through calibration. An initial stock solution of 1000 μg/mL of insulin using phosphate buffer solution was prepared. We prepared 10 mL of 6 different concentrations of 5, 10, 15, 20, 25 and 30 μg/mL from stock solution using the working PBS solution (pH = 1.2 or 6.8 or 7.4), and then a calibration plot was established at the maximum absorption wavelength (λ_max_ = 275 nm). The linear regression was found to be *y* = 0.0069*x* + 0.0071, where *y* corresponds to absorbance and *x* to the concentration of the released drug (μg/mL). A linear calibration plot with a correlation coefficient of 0.993 was used to quantify insulin release from the nanoformulations. Each experiment was performed in triplicates.

### 2.5. Cytotoxicity of Insulin/Ferrisilicate Nanoformulation against HFF-1 Cells

Human foreskin fibroblast (HFF-1) cells were obtained as (SCRC-1041TM, ATCC, Manassas, VA, USA) and maintained in DMEM supplied with 10% fetal bovine serum, 1% L-glutamine and 1% penicillin-streptomycin (Gibco, Thermo Fisher Scientific, Waltham, MA, USA) in a humidified 5% CO_2_ incubator (Galaxy^®^ 170S, Eppendorf, Stevenage, UK) at 37 °C. The cells were seeded in a 96-well plate at (10^4^ cells/well) and treated with group (a), insulin/ferrisilicate/PEG, insulin/10 wt% Fe/KIT-6/PEG, ferrisilicate/PEG and 10 wt% Fe/KIT-6/PEG using 25, 50, 100, 200, 400 and 800 μg/mL, and group (b), free insulin at 12.5, 25, 50, 100, 200 and 400 μg/mL, for 24, 48 and 72 h, including (c) untreated cells as control cells in each experiment. Both groups of concentrations are labeled as 1, 2, 3, 4 and 5 and were applied corresponding to the functionalized encapsulated insulin. MTT assay was performed to determine the cytotoxicity of the ferrisilicate nanoformulations by using a colorimetric-based reaction using MTT reagent 3-(4,5-dimethylthiazol-2-yl)-2,5-diphenyltetrazolium bromide to assess the metabolic activity of the cells. For the assay, 10 μl of MTT reagent (98%, 2128-1G, Sigma-Aldrich Chemie Holding GmbH, Taufkirchen, Germany) was added to obtain a final dilution of 1:10 and incubated for 4 h in a CO_2_ incubator at 37 °C. The formed formazan blue dye was solubilized by adding 100 μl of DMSO (Dimethyl sulfoxide) and read at 570 nm wavelength by a SYNERGY Neo2 multi-mode microplate reader (BioTek Instruments, Winooski, VT, USA). Cell viability was calculated as:

$$\mathrm{Cell}\;\mathrm{viability}\;\left(\%\right)=\left(\begin{array}{c} \underline{\mathrm{Absorbance}\;\mathrm{of}\;\mathrm{Sample}}\\ \mathrm{Absorbance}\;\mathrm{of}\;\mathrm{Control} \end{array}\right)\times 100$$

### 2.6. Statistics

The cytotoxicity assay was performed in three independent experiments. Statistical analysis was performed using Prism 9 software (GraphPad, La Jolla, CA, USA). The analysis was performed using two-way ANOVA with Dunnett’s multiple comparison test. Statistically non-significant p values were indicated as (ns). The data analysis of drug delivery was conducted using Prism 8 software and SPSS software version 20.0 (IBM Corp., Armonk, NY, USA).

### 2.7. In Vivo Study

Wistar rats of either sex weighing 180–200 g were used for the experiments. The animals are obtained from the IRMC animal house and were maintained and treated according to the IACUC policy. The study was approved by Imam Abdulrahman Bin Faisal University IRB through IRB number IRB-2020-13-278 with approval date of 30 September 2020. A single-dose study was performed to learn the effect of testing samples in diabetic rats using acute administration. The diabetic rats were divided into three groups, as follows:

Experimental Grouping:

Group 1: Diabetic rats orally administrated with normal saline (marketed dehydration fluid and electrolyte replenishment of sodium chloride) without the nanoformulation;

Group 2, 3 and 4: Diabetic rats treated with nanoformulations (orally administered) at three gradient doses of 2, 5 and 10 IU/kg body weight, respectively.

Blood glucose levels were measured at the start of the study (will be considered as initial blood glucose level) for all the animals included in the study. The glucose solution (2 g/kg) was administered orally 30 min after the administration of the testing samples, and blood samples were collected at 1, 2, 3, 4, 5 and 6 h after glucose administration to estimate the blood glucose levels.

## 3. Results and Discussion

### 3.1. Characterization of Ferrisilicate/Insulin/PEG

**Figure 1:**
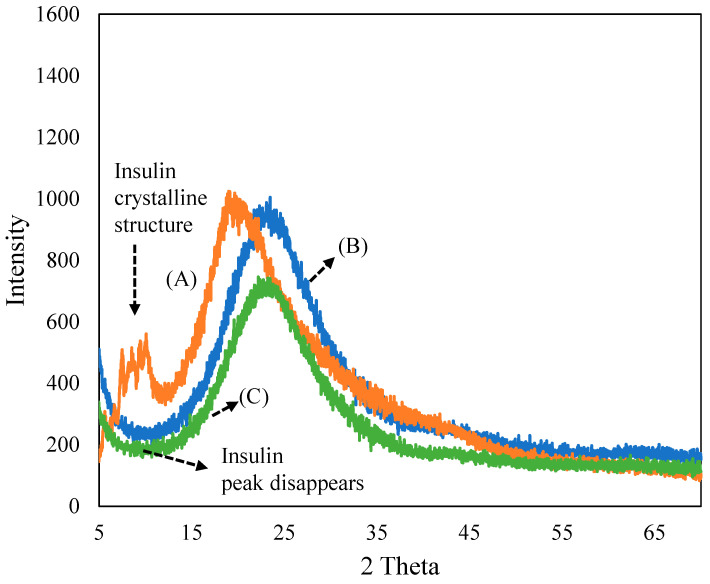
X-ray diffraction pattern of (**A**) insulin, (**B**) ferrisilicate and (**C**) insulin/ferrisilicate/PEG.

Figure 1 depicts the XRD patterns of (A) insulin, (B) ferrisilicate and the (C) insulin/ferrisilicate/PEG nanoformulation. The recombinant insulin powder exhibited a crystalline peaks (2θ range 5–15°), characteristic of macrostructured protein. The ferrisilicate exhibited a broad amorphous silica peak between 10 and 40°. In the insulin/ferrisilicate/PEG nanoformulation, the crystalline peak of insulin is absent, which clearly indicates the molecular dispersion or amorphous transformation of ferrisilicate.

**Figure 2:**
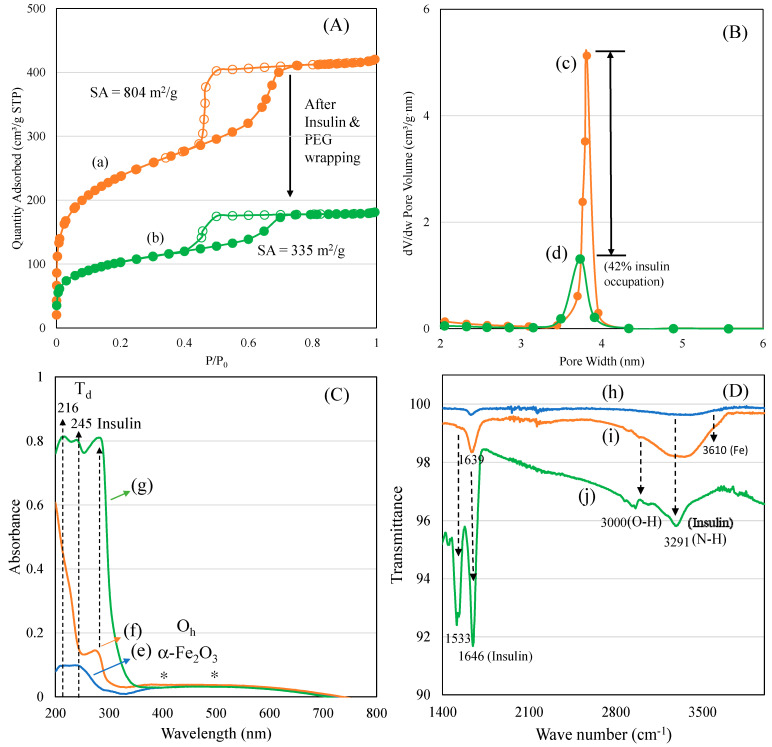
(**A**) Nitrogen adsorption isotherm: (a) ferrisilicate and (b) insulin/ferrisilicate/PEG. (**B**) Pore size distributions: (c) ferrisilicate and (d) insulin/ferrisilicate/PEG. (**C**) Diffuse reflectance spectra of (e) ferrisilicate, (f) insulin/ferrisilicate/PEG and (g) insulin. (**D**) FTIR spectra of (h) ferrisilicate, (i) insulin/ferrisilicate/PEG and (j) insulin.

A nitrogen adsorption isotherm was used to characterize the surface texture and pore diameter of mesoporous materials in the range of 2–50 nm. In our case, the changes in the ferrisilicate’s surface texture and three-dimensional cubic pores before and after insulin and PEG modifications were analyzed (Figure 2A,B). Parent ferrisilicate with an *Im3m* space group in the calcined form exhibited a type IV isotherm pattern with surface area of 804 m^2^/g, pore volume of 0.65 cm^3^/g and pore size centered at about 3.2 nm. After insulin loading and PEG wrapping, the quantity of nitrogen adsorbed reflected in the peak height that steeply decreased with capillary condensation (Figure 2A(a,b)). The hysteresis loop slightly decreases, indicating the occupation of pores by insulin. In parallel, the surface area (335 m^2^/g) and pore volume (0.28 cm^3^/g) decreases while the pore size stays at about 3.3 nm (Figure 2B(c,d)).

The textural changes in the samples before and after insulin/PEG wrapping were analyzed using nitrogen adsorption isotherm, and the results are presented in Table 1. A systematic change in the surface and pore volume indicates a successful insulin inclusion into the cubic pores of the ferrisilicate, while PEG wrapping occupies about 42% of the area around the 3D ferrisilicate. FeKIT-6 and mesosilicalite exhibited a similar high surface area and porous architecture. Overall, the textural modification indicates the insulin deposition in cubic cage pores of ferrisilicate (Figure 2A,B). The diffuse reflectance UV-visible spectra of ferrisilicate, insulin, and insulin/ferrisilicate/PEG are shown in Figure 2C(e–g). Ferrisilicate indicates a broad adsorption band in the 200–300 nm range, with peak maxima at 216 and 245 nm. The presence of a high-energy band is ascribed to the ligand-to-metal charge transfer due to tetrahedral Fe^3+^ species [22]. The band at 216 nm and 245 nm is ascribed to th(e electronic transition of O^2−^ to t_2g_ and e_g_ orbitals of Fe^3+^ in the iron oxide cluster. Unlike iron oxide-loaded mesoporous silica, ferrisilicate also shows a broad unresolved absorption band expanding between 400 and 500 nm. The absorption at 400 and 500 nm are ascribed to the quantum size effect of alpha-Fe_2_O_3_ species. Ferrisilicate shows the presence of Fe^3+^ species and the presence of a few hexacoordinated alpha-Fe_2_O_3_ species (Figure 2C(e)). Such iron oxide species occur in aggregated form, with octahedral or distorted octahedral coordination [23], inside the cubic pore channels of ferrisilicate. The insulin binding ability of ferrisilicate was measured using DRS-UV-Visible spectra. Insulin showed a strong absorption band at about 285 nm. The administering of ferrisilicate loaded with insulin (50 wt/wt%) followed by washing showed a similar peak to that of insulin at about 281 nm. The PEG wrapping shows effective conjugation with insulin through hydrogen bonding (Figure 2C(f,g)). Hinds et al. (2002) [24] stated that the PEG hydroxyl group interact with the amine functional moiety of the aminoacids of insulin. The FTIR spectra of ferrisilicate, insulin/ferrisilicate/PEG and insulin are shown in Figure 2D(h–j). Ferrisilicate exhibited characteristic peaks at the hydroxyl region between 3730 cm^−1^ and 3610 cm^−1^. The presence of a broad band at about 3610 cm^−1^ is attributed to the hydroxyl group bridging the signalling between the Bronsted sites related to the isomorphous substitution of Fe for Si in the ferrisilicate framework [25]. Insulin showed characteristic carbonyl (C=O) stretching vibration bands due to the presence of amide at 1646 cm^−1^ and 1533 cm^−1^. In case of insulin/ferrisilicate/PEG, the characteristic insulin vibration bands appear at about 1639 cm^−1^ and 1518 cm^−1^. An increase in such vibrational peaks clearly shows the coupling of insulin with ferrisilicate. Moreover, there is a shift in the main vibration bands of insulin occurring from 1646 cm^−1^ to 1639 cm^−1^. Such a shift in bands after the functionalization of insulin indicates the effective interaction between the insulin and ferrisilicate [26]. The loading capacity and entrapment of insulin inside the pore channels play a critical role in its release and anti-diabetic activity. Elongation in the broad hydroxyl (3000 cm^−1^) and amine stretching (3291 cm^−1^) bands compared to ferrisilicate indicates the effective functionalization of insulin in the insulin/ferrisilicate/PEG nanoformulation.

**Figure 3:**
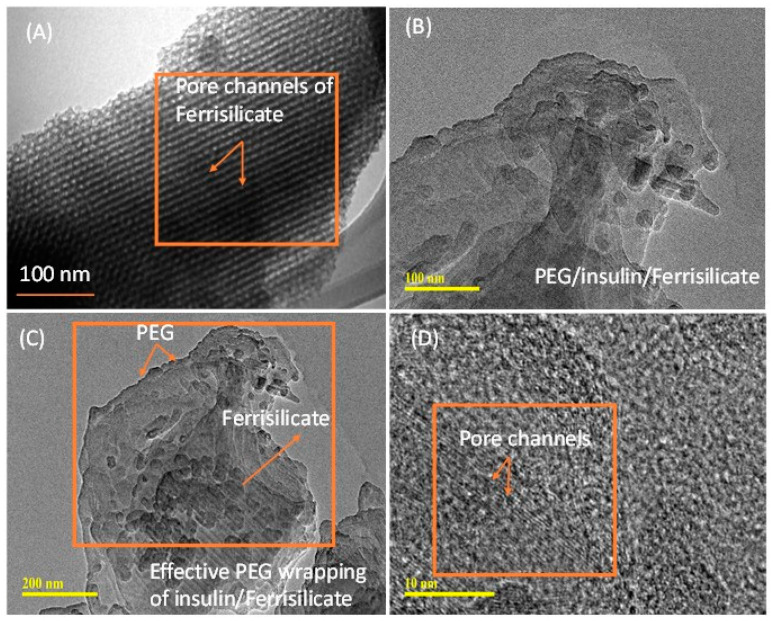
Transmission electron microscope images of (**A**) ferrisilicate and (**B**–**D**) insulin-loaded, PEG-wrapped ferrisilicate.

Figure 3A–D shows the morphological analyses of ferrisilicate and insulin-loaded, PEG-coated ferrisilicate. Ferrisilicate exhibits clear pore channels running on the parallel axis (Figure 3A). The analysis of the insulin-loaded, PEG-wrapped sample clearly shows the occurrence of structural transformation and enveloping by the polymeric layers. This indicates successful insulin loading and wrapping by PEG. In addition, magnification to 10 nm shows the presence of pore channels without any major changes inside the ferrisilicate (Figure 3C,D).

**Figure 4:**
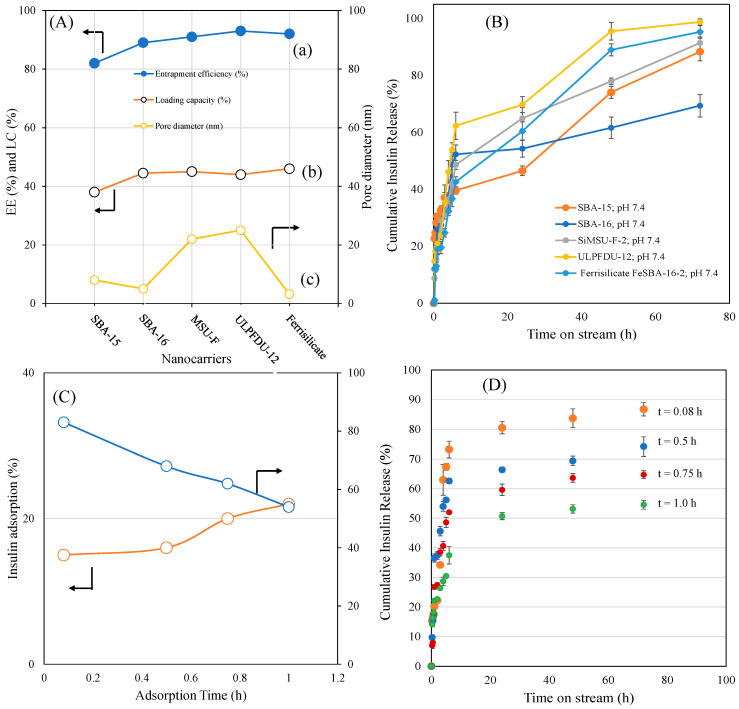
(**A**) Effect of different porous structured nanoformulations on (a) entrapment efficiency, (b) loading capacity and (c) pore diameter. (**B**) Percentage cumulative insulin delivery from different structured silicas. (**C**) Effect of different insulin adsorption times (t = 0.08 h, 0.5 h, 0.75 h and 1.0 h) on ferrisilicates versus percentage cumulative insulin release measured at 72 h. (**D**) Effect of insulin release (0.25–72 h) on ferrisilicate using different insulin adsorption times (0.08–1.0 h).

The effect of different porous-structured nanoformulations, entrapment efficiencies, loading capacities and insulin adsorption times (t = 0.08 h, 0.5 h, 0.75 h and 1.0 h) on insulin release are shown in Figure 4A–D. The insulin entrapment efficiency of hexagon-shaped SBA-15, cubic-shaped SBA-16, mesocellular forms with large pore windows, ultralarge-pore FDU-12 and ferrisilicate were studied. MSU-F and ULPFDU-12 contain large pores of 22 nm and 25 nm, respectively. SBA-15, SBA-16 and ferrisilicate have pore sizes of 8 nm, 5 nm and 3.2 nm, respectively. The entrapment efficiency was in the range of 82–93%, while the loading capacity was in the range of 38–46%. Interestingly, ferrisilicate showed high entrapment (92%) efficiency compared to large-pore nanomaterials MSU-F (91%) and ULPFDU-12 (93%). SBA-15 showed a slightly lower entrapment efficiency of 82% (Figure 4A). The insulin release profiles of encapsulated samples were studied using the dialysis membrane technique (Figure 4B). As expected, in the absence of polymer wrapping, the samples showed a high percentage cumulative insulin release. SBA-16, in the absence of iron species, showed a lower release of 68% at 72 h. The introduction of iron into SBA-16 silica (ferrisilicate) improves the insulin release by 95% compared to its silica counterpart, SiSBA-16. The presence of large pores favors the high release profile for MSU-F (91%), ULPFDU-12 (98%) and SBA-15 (91%).

The effect of insulin adsorption over different adsorption times (t = 0.08 h, 0.5 h, 0.75 h and 1.0 h) on insulin release is shown in Figure 4C,D. Ferrisilicate shows an increase in insulin adsorption with time (15.7% in 0.08 h, 18.6% in 0.5 h, 20.5% in 0.75 h and 22% within 1 h). This result demonstrates that a higher adsorption time reduces insulin release. It indicates an effective entrapment with higher adsorption time. The entrapment efficiency and loading capacity reveals the efficiency of the drug-loaded nanoformulation [27]. A glucose-responsive system has been reported from PBA containing structured silica coated with diol-based copolymers (*N*-acryloyl glucosamine and *N*-isopropyl acrylamide). The formulation showed a high loading capacity (14.7%) and encapsulation efficiency (85.9%) with glucose responsive release at pH = 7.4 [28]. It has been reported that PBA- and diol-based block copolymers along with post-modification improved the glucose-sensitive release of insulin [29]. Replacing PBA with fluorophenylboronic acid has been reported to improve insulin loading, resulting in a high encapsulation efficiency and better glucose responses in physiological conditions [30]. Glucose-responsive sulfonamide–PBA, with its temperature-responsive properties, was reported to improve both loading capacity and encapsulation efficiency. These nanoparticles are reported to be safe and effective for the subcutaneous injection of insulin [31]. In our case, the entrapment efficiency was 92%, while the drug loading capacity reached up to 46%. 

**Figure 5:**
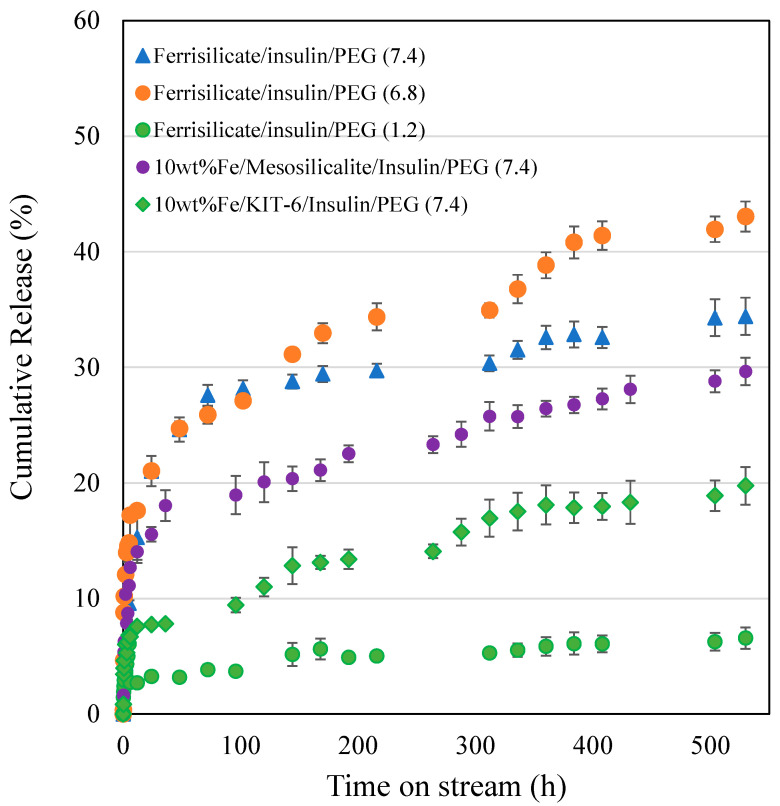
Insulin release by pegylated ferrisilicate, 10 wt% Fe/mesosilicalite and Fe/KIT-6 at different pH conditions (7.4, 6.8 and 1.2). The drug release experiment was performed in triplicates.

The insulin release over pegylated ferrisilicate, 10 wt% Fe/mesosilicalite and Fe/KIT-6 at different pH conditions (7.4, 6.8 and 1.2) are studied for 530 h (Figure 5). Ferrisilicate displays a high percentage of cumulative release of insulin of about 40–50%. The 10 wt% Fe/mesosilicalite and Fe/KIT-6, which contain 3D pore architectures, showed a release of about 20–30% over 530 h. This indicates the ink-shaped pores of SBA-16 (about 3.3 nm) are slightly restricted with Fe impregnation, showing a sustained release behavior with respect to insulin. The presence of 3D cage-type pores with Ia3d structures in KIT-6 was found to favor the slow release of insulin (20% for 530 h), while mesosilicalite with hexagonal pores of MCM-41 showed a release of about 30% over 530 h. This suggests that insulin tends to functionalize on the external micropores of mesosilicalite, while cage-type pores are able to accommodate the insulin inside the mesopores. Polysaccharide pullulan hydrogel in the form of carboxylation was reacted with concanavalin A (Con A) using amidization reaction. The nanoformulation was shown to control the release of insulin due to the specific bonding occurring between protein andglucose binding and to the crosslinked structure with uniform pores that accommodates insulin [32]. A detailed study on the glucose-responsive insulin release behavior of hydrogel/microgels-Con A nanoformulations shows that the release trend is controlled by the bolus and basal insulin release and network composition [33]. Konjac Mannan (heteropolysaccharide) fabricated with Con A through the crosslinking technique exhibited a targeted insulin release. The nanoformulation with a particle size of about 500 nm facilitated a glucose-responsive insulin release with a reversible pattern with different levels of glucose. Furthermore, the in vivo study reveals that the nanoparticles are non-toxic and able to control the blood sugar level for 6 h [34]. An immobilization of glucose oxidase on a biocompatible linear polysaccharide alginate/phenylboronic acid-derived composite exhibited an improved glucose sensitivity with a potential option for subcutaneous insulin delivery [35]. However, the protein stability, antigenicity and synthesis cost limit the design of such protein-based nanoformulations for clinical translation [36,37]. In case of phenylboronic acid functionalization, the cytotoxicity and lower solubility due to higher pKa (~9.0) are some limitations [38]. In the present study, the facile and simple nanocomposite formation between ferrisilicate and PEG can perform similar pH-sensitive insulin release.

### 3.2. Kinetics of Different Ferrisilicate/Insulin Nanoformulation Drug Release Using the Korsmeyer–Peppas Model

The different ferrisilicate/insulin nanoformulation drug release profiles at different pHs were examined using the Korsmeyer–Peppas model, expressed using the equation:

$$R\;\%={\mathrm{kt}}^{n}$$

where R% is the different ferrisilicalite/insulin drug percentage release at time (t) and k and n are the kinetic release rate constant and the release exponent, respectively.

The kinetic parameters with their 95% confidence intervals are presented in Table 2.

For all the base materials; SiSBA-15, SiSBA-16, SiMSU-F and SiULPFDU-12, the rate of drug release is enhanced as observed from the higher release constants which are all higher than that of the modified FeSBA-16 base material. Similarly, the release mechanisms of the drugs nanoformulation obtained from the base materials followed the fickian diffusion mechanism (n < 0.45), while the modified FeSBA-16 drug release followed the non-fickian (0.45 < 0.488 < 0.89) diffusion mechanism. These indicate that modifying the base materials affect both the rate of release and the release diffusion mechanism.

The effect of four different insulin adsorption times (5, 30, 45 and 60 min) on the rate of release and diffusion mechanism of ferrisilicate nanoformulation drug shown that, the drug release mechanism at all the insulin adsorption times followed the fickian (n < 0.45) diffusion. However, the rate of drug release as signified by the released constant revealed that, the rate of drug release is highest after 30 min of insulin adsorption. Also, there is no direct correlation between the adsorption times studied and the rate of drug release, because the rate increased up to a maximum at 30 min adsorption, then continued to decline at higher insulin adsorption. This indicates that, there is a saturation limit of insulin adsorption, above which the release rate of the drug is affected.

Different insulin/ferrisilicate ratios of 0.125, 0.25, 0.75 and 1.0 were utilized in the nanoformulation of drugs. The rate of drug release is highest at higher ratio of 1.0, indicating that the higher the insulin/ferrisilicate ratio, the greater the rate of drug release, except at the ratio of 0.75. The nanoformulation at all ratios followed the fickian diffusion mechanism. This confirmed that, the kinetic release profile of the nanoformed insulin/ferrisilicate drug is affected by the ratio of the insulin to the ferrisilicate base material.

The effect of different pH of ferrisilicate on the diffusion mechanisms and rate of ferrisilicate/insulin/PEG drug release revealed that, at close to neutral pH of 6.8, the rate of drug release is highest relative to highly acidic (pH = 1.2) and slightly basic (pH = 7.4) conditions. However, at both pH of 1.2 and 6.8, the release exponent signified fickian diffusion mechanism, while at pH of 7.4, the release mechanism changed to non-fickian diffusion. For these nanoformulation, it can be inferred that the pH of ferrisilicate affects both the rate of drug release and the diffusion mechanism.

Four drug nanoformulation with different base materials modification of ferrisilicate but having the same PEG coating at the same pH revealed that, the rate of drug release is enhanced with the modification, because of their higher release constant compared to the unmodified ferrisilicate. Also, the diffusion mechanism is affected by the modification as follows; 10 wt% Fe/KIT-6 and 10 wt% Fe/Mesosilicalite based nanoformulation drugs release followed the fickian (n < 0.45) diffusion mechanism while unmodified ferrisilicatefollowed the non-fickian (0.45 < n < 0.89) diffusion mechanism. 

For the null hypothesis: there is no significant differences between the various release exponent and 0.45, one-paired t-test was carried out and the calculated *p*-value (0.00003736) which is <<0.05 showed that the null hypothesis is invalid, rather the alternative hypothesis: that there are significant differences between the release exponents (n) and 0.45 is valid.

### 3.3. In Vitro Study

**Figure 6:**
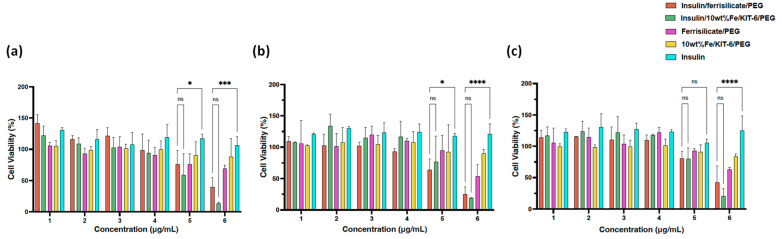
Cytotoxicity of Insulin/Ferrisilicate/PEG nanoformulations against Human foreskin fibroblast (HFF-1) cells. The cells were treated with Insulin/Ferrislicate/PEG, Insulin/10 wt% Fe/KIT-6/PEG, Ferrisilicate/PEG, 10 wt% FeKIT-6/PEG and insulin for (**a**) 24, (**b**) 48 and (**c**) 72 h. Error bars ± SD. (ns) non-significant *p* value, * *p* < 0.05; *** *p* < 0.001; **** *p* < 0.0001 as Insulin/Ferrisilicate/PEG versus Insulin/10 wt% Fe/KIT-6/PEG and insulin using two-way ANOVA with Dunnett’s multiple comparison test. The cytotoxicity assay was performed in three independent experiments.

The cytotoxicity of ferrisilicate nanoformulations were studied on Human foreskin fibroblast (HFF-1) cells (Figure 6). Insulin/Ferrisilicate/PEG and insulin/10 wt% Fe/KIT-6/PEG were obliviously showing no cytotoxicity at 25, 50, 100, 200 μg/mL after each timepoint even after 72 h, and started to show cytotoxic effect by less than 50% cell viability at the highest concentration 800 μg/mL. Ferrisilicate, insulin/10 wt% Fe/KIT-6 and insulin were used as control groups to assess the cytotoxicity of empty vectors or NP material, as shown ferrisilicate and 10 wt% Fe/KIT-6 were not toxic even at the highest concentration 800 μg/mL post 72 h of treatment. Expectedly, insulin has stimulated cell growth to reach to 130% in comparison to DMEM- treated cells which have a 100% cell viability and used as control values in this experiment. 

### 3.4. In Vivo Study

**Figure 7:**
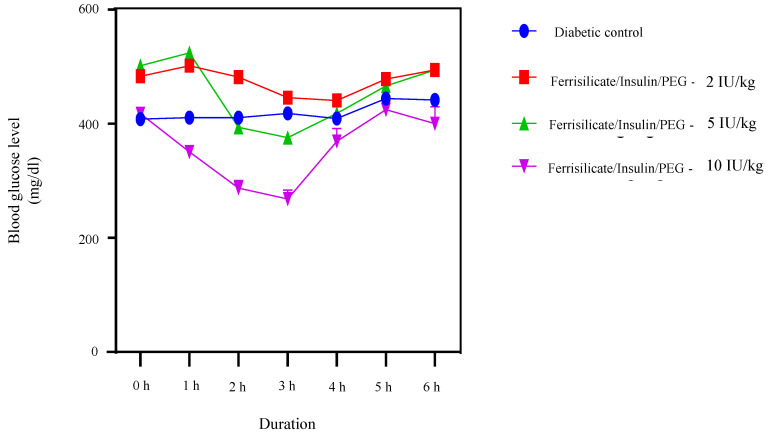
Hypoglycemic effect of insulin/ferrisilicate nanoformulation Insulin/Ferrisilicate/PEG administered to the diabetic animals at three gradient doses 2, 5, and 10 IU/kg body weight (n = 6).

To find out the hypoglycemic effect of the prepared insulin nanoformulation, it was administered orally to the diabetic animals at three gradient doses 2, 5 and 10 (Insulin Unit) IU/kg body weight (Figure 7). The blood glucose level was measured at different intervals up to 6 h. The results of the study indicate the hypoglycemic effect of insulin nanoformulation at a dose of 5 and 10 IU/kg, and the formulation was found to be effective after 2 h of administration at a dose of 5 mg/kg. Furthermore, it reduced the blood glucose level significantly (*p* < 0.0001) until 3 h, after which it starts to move towards the initial blood glucose values. At 5 IU/kg, the insulin/ferrisilicate/PEG nanoformulation decreases the blood glucose level from 501 to 375 mg/dL (reduction of 25%). Moreover, the formulation at 10 IU/kg body weight showed a significant reduction (*p* < 0.0001) in blood glucose level after 1 h of administration and was found to be significant until 3 h of administration. It reduced the blood glucose level from 417 to 268 mg/dL (35% reduction). These effects of the nanoformulation at a dose of 5 and 10 IU/kg body weight were found to be significant (*p* < 0.0001), as compared to diabetic control. No significant effect of insulin formulation was found at 2 IU/kg body weight.

## 4. Conclusions

In the present investigation, pegylated 3D cubic porous ferrisilicate was explored for diabetic management. The physico-chemical characterization reveals the incorporation of Fe^3+^ species into the framework (via isomorphous substitution) of SBA-16. A large surface area and pore size were found to be sufficient for a high encapsulation of insulin (46%) at 1.5 h. The adsorption increases with time to about 57% at 1.5 h. Ferrisilicate exhibited a high percentage of cumulative insulin release of about 32% in 530 h at pH 7.4. Insulin release was notably improved by PEG wrapping (0.08 μL/mg) to about 41% in 530 h at pH 6.8. As a comparison, Fe/Mesocellular foam, Fe/Mesosilicalite and Fe/KIT-6 showed about 40%, 28% and 19.2% insulin release in 530 h. The kinetic studies using the Korsmeyer–Peppas model revealed that the nature of the base materials, different insulin adsorption times, varying insulin/ferrisilicate ratios, different pHs and different base material modifications all affect the rate of insulin release and its diffusion mechanism. The nanoformulation showed very low toxicity in in vitro study and a hypoglycemic effect in in vivo study. Doses of 5 and 10 mg/kg body weight of pegylated ferrisilicate were found to be significant (*p* < 0.0001) compared to the diabetic control. Overall, the developed 3D porous ferrisilicate wrapped with polyethylene glycol mimics smart stimuli-responsive behavior with a pH-sensitive insulin release with high cell viability for potential application in diabetic management.

## Figures and Tables

**Table 1:** Textural properties of insulin and PEG-wrapped nanoformulations.

Nanocarriers	BET Surface Area (m^2^/g)	Pore Volume (cm^3^/g)	Average Pore Size (nm)
Ferrisilicate	804	0.65	3.2
Insulin/Ferrisilicate/PEG	335	0.28	3.3
Insulin/Fe-Mesosilicalite/PEG	360	0.38	4.2
Insulin/Fe-KIT-6/PEG	560	0.84	5.9

**Table 2:** Kinetic parameters for insulin release from different porous-structured nanoformulations. Influence of adsorption time, insulin-to-ferrisilicate ratios and release at different pH conditions.

Drug Formulations	k (h^−n^)	n	n−0.45	R^2^
				
SBA-15; pH 7.4	25.2086 ± 3.4391	0.2671 ± 0.0596	0.1829	0.9088
SBA-16; pH 7.4	21.9335 ± 3.2175	0.2882 ± 0.0638	0.1618	0.9102
MSU-F; pH 7.4	18.0398 ± 2.4209	0.4153 ± 0.0587	0.0347	0.9613
ULPFDU-12; pH 7.4	18.0954 ± 2.9981	0.4337 ± 0.0714	0.0163	0.9482
Ferrisilicate FeSBA-16; pH 7.4	13.7013 ± 2.1190	0.4879 ± 0.0670	0.0379	0.9634
				
				
Ferrisilicate; Adsorp time = 5 min	23.5404 ± 4.6002	0.3746 ± 0.0832	0.0754	0.9097
Ferrisilicate; Adsorp time = 30 min	27.6661 ± 5.4414	0.2840 ± 0.0837	0.1660	0.8512
Ferrisilicate; Adsorp time = 45 min	21.1912 ± 3.7614	0.3351 ± 0.0761	0.1149	0.9058
Ferrisilicate; Adsorp time = 60 min	20.1574 ± 1.2871	0.2555 ± 0.0288	0.1945	0.9750
				
				
Insulin/Ferrisilicate ratio 0.125-11-7.4	21.6909 ± 2.1147	0.2558 ± 0.0507	0.1942	0.9353
Insulin/Ferrisilicate ratio 0.25-12-7.4	26.0871 ± 3.4667	0.2814 ± 0.0680	0.1686	0.9067
Insulin/Ferrisilicate ratio 0.75-13-7.4	21.9251 ± 4.1966	0.4021 ± 0.0955	0.0479	0.9097
Insulin/Ferrisilicate ratio 1.0-14-7.4	36.3453 ± 3.6093	0.2102 ± 0.0516	0.2398	0.9040
				
				
Ferrisilicate/insulin/PEG (7.4)	1.4153 ± 0.5882	0.5722 ± 0.0837	0.1222	0.9013
Ferrisilicate/insulin/PEG (6.8)	7.8563 ± 1.3468	0.2728 ± 0.0381	0.1772	0.9093
Ferrisilicate/insulin/PEG (1.2)	2.6017 ± 0.1705	0.1791 ± 0.0151	0.2709	0.9631
				
				
10 wt% Fe/Mesosilicalite/insulin/PEG (7.4)	5.4089 ± 0.9689	0.2771 ± 0.0385	0.1729	0.9016
10 wt % Fe/KIT-6/insulin/PEG (7.4)	3.7004 ± 0.4118	0.2533 ± 0.0241	0.1967	0.9490

## Data Availability

The data sets, figures and analyses are provided in this manuscript. Further additional data will be available from the corresponding author upon request.
